# The history of the North African mitochondrial DNA haplogroup U6 gene flow into the African, Eurasian and American continents

**DOI:** 10.1186/1471-2148-14-109

**Published:** 2014-05-19

**Authors:** Bernard Secher, Rosa Fregel, José M Larruga, Vicente M Cabrera, Phillip Endicott, José J Pestano, Ana M González

**Affiliations:** 1 Administrator of U6 mtDNA project at Family Tree DNA, Houston, TX, USA; 2 Department of Genetics, Faculty of Biology, Universidad de La Laguna, La Laguna, Tenerife, Spain; 3 Department of Genetics, Faculty of Medicine, Universidad de Las Palmas de Gran Canaria, Las Palmas de Gran Canaria, Gran Canaria, Spain; 4 UMR 7206 Eco-anthropologie. Equipe “génétique des populations humaines”, Musée de l’Homme. CP139. 61 rue Buffon, 75005 Paris, France; 5 Forensic Genetics Laboratory, Institute of Legal Medicine of Las Palmas, Las Palmas de Gran Canaria, Gran Canaria, Spain

**Keywords:** Population genetics, Human evolution, Mitochondrial DNA, Haplogroup U6, Phylogeny, Phylogeography

## Abstract

**Background:**

Complete mitochondrial DNA (mtDNA) genome analyses have greatly improved the phylogeny and phylogeography of human mtDNA. Human mitochondrial DNA haplogroup U6 has been considered as a molecular signal of a Paleolithic return to North Africa of modern humans from southwestern Asia.

**Results:**

Using 230 complete sequences we have refined the U6 phylogeny, and improved the phylogeographic information by the analysis of 761 partial sequences. This approach provides chronological limits for its arrival to Africa, followed by its spreads there according to climatic fluctuations, and its secondary prehistoric and historic migrations out of Africa colonizing Europe, the Canary Islands and the American Continent.

**Conclusions:**

The U6 expansions and contractions inside Africa faithfully reflect the climatic fluctuations that occurred in this Continent affecting also the Canary Islands. Mediterranean contacts drove these lineages to Europe, at least since the Neolithic. In turn, the European colonization brought different U6 lineages throughout the American Continent leaving the specific sign of the colonizers origin.

## Background

Easy detection and the haploid characteristics of mitochondrial DNA (mtDNA) make this molecule an ideal tool for studies of human evolution and dispersion [1]. In spite of the caution required in inferring human population history from the genealogy of a single locus, mtDNA has still been very successful to either reinforce or refute hypotheses on human evolution. Using mtDNA restriction polymorphisms, it was first proposed that all extant modern humans have a recent African origin [2]; a hypothesis that found physical anchorage in the paleoanthropological record [3,4].

After the first spread out of Africa, one of the most important modern human movements was a Paleolithic back-flow to Africa. Clear signals of this return were deduced from the phylogeny and phylogeography of the mtDNA haplogroups U6 [5-9] and M1 [5,7,8,10], which show major North and East African distributions. The genealogy and geographic distribution of at least two African branches of the West-Eurasian Y-chromosome haplogroups R and T (R-V88 and T-M70, respectively) [11-13], gave additional evidence for this back migration from a paternal perspective.

Primary and secondary radiations of U6 branches with different coalescence ages were tentatively correlated with different North African lithic cultures, such as the Aterian, Dabban, Iberomaurusian or Capsian; and perhaps more speculatively, with the spread of the Afroasiatic language family. The Aterian was thought to have existed between 40–20 kya but recent archaeological age determinations, based on thermal luminescence, have pushed back this period, to 90–40 kya [14-16]. As the estimated age for the whole of haplogroup U6 is around 35 kya, this removes the Aterian from consideration for association with the genetic signal for dispersal in North Africa [8,9]. However, as U6 persists in modern day African populations we can assume a maternal continuity since around 35 kya, the age of this haplogroup. This continuity has received some support from ancient DNA studies on Iberomaurusian remains, with an age around 12 kya, exhumed from the archaeological site of Taforalt in Morocco [17]. In this analysis, haplotypes tentatively assignable to haplogroups H, JT, U6 and V were identified, pointing to a local evolution of this population and a genetic continuity in North Africa. On the other hand, only one haplotype harbored the 16223 mutation, which if assigned to an L haplogroup would represent a sub-Saharan African influence of about 4%. This would equate to a frequency five times lower than that found in current Moroccan populations (20%) and would support the proposal that the penetration of sub-Saharan mtDNA lineages to North Africa mainly occurred since the beginning of the Holocene onwards [18].

It is possible that the substitution of old industries by new ones sometimes implied external gene flow, but not enough to totally replace the resident population. In this study we analyze 230 complete U6 sequences and 761 partial ones in order to investigate, first, the demographic evolution, inside Africa, of haplogroup U6 and, second, the age and most probable origin of the secondary spreads that carried U6 lineages to Europe and the Americas. In addition, we propose a model that might reconcile the genetic history of U6 with the extant paleoanthropological and archaeological records for the same period.

## Methods

### Samples

A stock of 375 U6 samples, previously identified in La Laguna, was subdivided into the following large geographic areas: Africa, Europe and the Middle East. Taking into account their relative numbers, 40 individuals were randomly chosen within each region for complete sequencing. In addition, 29 U6 individuals were contacted through the FTDNA U6 project and written consent obtained to use them in the current study. Maternal geographic origin, at least until the second generation, was known for each donor as detailed in Additional file 1. Only family members of the Acadian cluster were known to be related individuals. Written informed consent to anonymously use their DNA samples was obtained from all donors. This project was approved by the Ethics Commission of the University of La Laguna and complied with the Helsinki Declaration of Ethical Principles.

### DNA extraction, amplification and complete sequencing

DNA was extracted from buccal swabs or blood stains following a protocol based on the use of proteinase K, dithiothreitol and sodium dodecyl sulfate [19]. In order to avoid bacterial growth, buccal swabs, sent to the laboratory by mail, were packed into screw cap tubes with ethanol. In place and after alcohol evaporation, the same DNA extraction protocol was employed.

Complete mtDNA was amplified in 32 overlapping fragments with primers and PCR conditions previously described [5]. The same forward primers were used for sequencing one strand and, when necessary, the reverse was also employed so as to sequence both strands. Sequences in La Laguna were run on a MegaBase and in Las Palmas on an ABI 3130xl analyzer using the appropriate chemicals in each case. In addition, fourteen U6 previously published complete mtDNA genomes using P^32^[6] were re-analyzed and where necessary, some fragments comprising dubious positions, re-sequenced. In a few cases, old samples did not have enough DNA to securely amplify the fragments necessary to review those dubious positions. For these cases we performed a genomic amplification using the GenomiPhi DNA Amplification kit (GE Healthcare Life Sciences), following instructions provided with the kit.

Sequence data were aligned and assembled with BioEdit [20] and SeqScape software programs, respectively. All chromatograms were visually inspected in both laboratories. Nomenclature was as in van Oven and Kayser (mtDNA tree Build 15; 30-9-2012) [21]. GenBank accession numbers for all the sequences are detailed in Additional file 1.

### Data analyses

In addition to our 69 sequences, we used another 161 U6 complete sequences, previously published or available in GenBank (see Additional file 1), to construct the most parsimonious U6 phylogenetic tree [22], by means of Network 4.6 software, and further refined by hand (see Additional file 2). Coalescence ages for the total U6 phylogeny, and for each of its subgroups, were estimated using the mutation rate (one every 3624 years) and calculator provided by Soares et al. [23]. Accompanying standard errors were calculated as per Saillard et al. [24].

To depict the U6-inferred female effective population size through time we obtained Bayesian skyline plots using the BEAST software [25] version 1.6.2 (http://beast.bio.ed.ac.uk) and conditions described before [26]. For this purpose, we chose to apply a strict molecular clock with the same mutation rate used to estimate coalescences. The results were visualized with Tracer v1.5 (http://tree.bio.ed.ac.uk/software/tracer).

Frequency distributions of haplogroup U6 and its main subhaplogroups, based on HV1 sequences, were graphically visualized by contour maps created by the Kriging method, using the Surfer version 9.11.947 (Golden Software Inc). Principal Component Analysis (PCA) was performed on HV1-based U6 subgroup frequencies using the IBM SPSS Statistics 19 version, software package. Gene diversity was calculated as implemented in Arlequin 3.5.1.2 software [27].

## Results

### U6 phylogeny

Fourteen previously published U6 sequences using P^32^[6] have been reanalyzed. After careful re-reading and partial re-sequencing we detect that sequences AF382008, AY275531, and AY275532 all have 794A transversion and 1193 transition; AY275527 has 4062, 12535 and 13637 transitions; AY275533 has 12950C transversion; AY275536 and AY275537 have 3688C transversion and 13879 transition; AY275535 has the mutations 143, 750!, 8282 and 10172; and transition 2109 has been removed from AY275536.

Additional file 2 shows the U6 phylogenetic tree based on 230 complete sequences. Although the main branches have been described previously [6-9], this enlarged sequence data-set allows us to considerably refine the U6 phylogeny. Compared to the PhyloTree.org Build 15 phylogeny [21] and U6 tree [8], within U6a1 two new sub-groups U6a1a1a2 (13071) and U6a1b1b (2158, 10336, 14034, 16145) are identified. Likewise, within U6a2a two new nested sub-groups U6a2a3 (transitions 4936, 9100, 9128, 10172, 16295 and transversions 5894C and 9335A) and U6a2a3a (15626 reversion) are detected. U6a2b is now characterized by transitions 15383 and 16354, whereas its subgroup U6a2b1 is defined by 15314 and 16184 transitions. A new U6a2 branch, U6a2c, is defined by transition 195. Within U6a3 six new sub-groups U6a3a1b (8598), U6a3b1a (16311), U6a3c (146, 291.1A, 960d, 1809, 5554A, 6182, 11272, 15380), U6a3e (185, 3337, 4021, 8705, 12097, 13569, 13928, 16362, 16399), U6a3f (150, 185, 310, 8763), U6a3g (150, 3826) are identified. The U6a8 sub-group, that shares 16189 with U6a2 and U6a3, is now defined by 143, 8282, 10172, 11539 transitions and 750 reversion. The U6a5a1 is now only diagnosed by 11191, so that the previous U6a5a1 [8] is renamed U6a5a1a. Within U6a5, a new U6a5b sub-group (3714, 16184, 16234) is identified. Transition 16079 is now a diagnostic mutation of U6a6a, and a new branch, U6a6a1, is defined by 9031. Transition 5120, included before in a string of 8 diagnostic mutations of haplogroup U6a7a, now defines the new sub-haplogroup U6a7a1. Transversion 12950C is included in the basal branch of U6a7b1. Finally, six new U6a7 sub-groups U6a7a1a (2672, 11929), U6a7a1b (150), U6a7a1c (152 reversion), U6a7a2a (14034), U6a7a2a1 (11941) and U6a7b1a (455.1 T, 960.1C, 11818C, 12940, 13879) are identified.

Within the Canary Islands specific U6b1a clade, now defined only by 2352 and 16163 positions [8], three new branches can be distinguished, U6b1a1, U6b1a2 and U6b1a3 defined by transitions 7700, 6734, and 15697 and 16092 respectively. This Canarian specific branch groups with the North African sister branch U6b1b, sharing substitutions 9738 and 15431 which define now the U6b1 clade [8]. In addition, at least four sister branches of U6b1 can now be identified: U6b2 (4062, 12535, 13637, 15355), U6b3 (16278), U6b4 (5442, 16051), and U6b5 (5773, 8951, 14053, 16111, 16362). Within U6b3, a new U6b3a sub-group is defined by transition 235. U6d3 is now only defined by transition 16174, so that the previous U6d3 [8] is now renamed U6d3a. Within U6c1 three branches, U6c1a (12406, 16111), U6c1b (16086) and U6c1c (5964, 12092A, 15617) are defined. A sister clade, U6c2, is now diagnosed only by transition 194 and its sub-group U6c2a by transition 3866. Other uncertain subdivisions will be considered only within their phylogeographic context.

As mentioned recently [9], phylogenetic classification of U6 haplotypes based solely on diagnostic positions in the hypervariable region 1 (HVR-1) can be misleading. However, in order to use an important dataset of 761 U6 HVR-1 sequences, extracted from a worldwide screening of 59,060 HVR-1 sequences (Table 1; Additional file 3), for phylogeographic purposes, we have sorted them into the following phylogenetic sub-groups: U6a (16278), joining haplogroups U6a5 and U6a7 that are distributed in an Atlantic range from Europe to West Africa; U6a (16278, 16235) and U6a (16189, 16278, 16239) that approximate to haplogroups U6a1b and U6a1a1 with a central-western Mediterranean range; U6a (16189, 16278), comprising haplogroups U6a2, U6a3 and U6a8 respectively, spreading across eastern and western areas of the Sudan belt; U6b (16311), a geographically widespread cluster with a subgroup U6b1a (16163) endemic to the Canary Islands; U6d (16311), represented by its subgroups U6d1 (16261) and U6d3 (16174), both of western Mediterranean adscription; and U6c (16169, 16189) present mainly in southern Italy (16111), and the Canaries (16129).

**Table 1:** U6 subhaplogroups

**Region**^**1**^			**MAG**	**MAG**	**EAF**	**MAG**	**COS**	**CAN**	**MAG**	**MED**	**References**^**2**^
**Population**^**3**^	**Sample size**	**%U6**	**U6a without 235**	**U6a 235**	**U6a189 without 239**	**U6a189 239**	**U6b**^**4**^	**U6b1**	**U6d**^**4**^	**U6c**	
**BRA**	1400	0.57	12.5	-	87.5	-	-	-	-	-	18, 92, 101, 107, 126, 151, 164, 170, 191
**HIS**^**5**^	4652	0.62	11.4	5.7	5.7	2.9	-	65.7	2.9	5.7	28, 31, 32, 42, 43, 47, 50, 51, 61, 88, 91, 98, 100, 116, 128, 133, 140, 146, 150, 177, 179, 181, 182, 183, 184, 201
**HUS**	1062	0.75	-	-	25.0	-	25.0	37.5	12.5	-	12, 47, 67, 117, 187, 216
**AUS**^**5**^	1938	0.62	14.3	-	78.6	7.1	-	-	-	-	11, 72, 76, 117, 192
**CUS**^**5**^	1959	0.31	12.5	-	50.0	31.3	6.3	-	-	-	10, 117, 132, 192
**POR**	1187	2.53	10.0	23.3	20.0	23.3	6.7	3.3	13.3	-	62, 93, 153, 154
**AZO**	471	2.55	33.3	-	16.7	8.3	8.3	-	33.3	-	40, 185, 186
**MAD**	155	3.23	60.0	-	20.0	-	-	-	20.0	-	40
**CAV**	323	3.10	20.0	-	70.0	-	10.0	-	-	-	39
**SPA**	4110	1.12	23.9	6.5	28.3	8.7	10.9	8.7	10.9	2.2	9, 15, 16, 17, 29, 49, 62, 65, 67, 77, 81, 83, 89, 90, 93, 118, 119, 122, 129, 159, 161, 162, 173, 178, 180, 216
**CAN**	1040	16.15	4.2	0.6	2.4	1.2	1.2	76.8	1.2	12.5	167, 185, 216
**NWE**	11409	0.11	16.7	-	33.3	16.7	16.7	-	-	16.7	13, 15, 22, 23, 37, 66, 73, 74, 89, 96, 103, 104, 105, 106, 121, 131, 138, 155, 156, 160, 163, 165, 171, 172, 173, 175, 176, 194, 196, 216
**MdC**	3680	0.41	33.3	-	46.7	6.7	-	-	13.3	-	8, 24, 30, 41, 46, 71, 81, 84, 85, 86, 87, 136, 139, 149, 157, 173, 195, 197, 204, 205, 207, 209, 211
**MdE**	8401	0.19	25.0	-	62.5	-	12.5	-	-	-	19, 20, 21, 24, 26, 27, 33, 36, 45, 59, 60, 68, 70, 71, 75, 84, 95, 108, 109, 113, 123, 124, 127, 135, 137, 141, 142, 143, 144, 148, 166, 173, 190, 198, 199, 200, 208, 213, 214, 215
**SAM**	284	8.10	56.5	-	30.4	8.7	4.3	-	-	-	94, 162, 168, 216
**MOR**	1508	8.89	30.6	6.0	37.3	9.7	9.7	-	2.2	4.5	15, 34, 63, 64, 81, 102, 162, 168, 169, 198, 203, 216
**ALG**	299	6.69	15.0	15.0	55.0	5.0	-	-	-	10.0	162, 216
**TUN**	951	5.36	33.3	-	39.2	19.6	2.0	-	-	5.9	57, 63, 78, 79, 120, 162, 203, 216
**NEA**	1081	1.57	23.5	-	35.3	23.5	17.6	-	-	-	64, 80, 114, 115, 147, 188, 193, 216
**EAF**	534	2.62	-	-	100.0	-	-	-	-	-	38, 112, 198, 212, 216
**ARP**	3224	1.09	11.4	34.3	22.9	-	31.4	-	-	-	1, 2, 3, 4, 5, 6, 7, 14, 55, 63, 112, 145, 189, 198
**WAF**	3471	1.47	15.7	-	64.7	-	15.7	-	3.9	-	39, 44, 48, 52, 53, 54, 56, 58, 69, 82, 97, 110, 130, 152, 161, 168, 174, 191, 192, 202, 206, 210, 212, 216
**CAF**	2099	0.43	33.3	-	44.4	-	22.2	-	-	-	52, 56, 76, 94, 152, 206, 212, 216
**JEW**	2860	0.52	26.7	-	73.3	-	-	-	-	-	25, 26, 35, 36, 145, 158, 159, 198
**GYP**	944	0.74	-	-	-	42.9	57.1	-	-	-	75, 83, 99, 111, 125, 134

Most probable geographic origins and their frequencies in the regions analyzed.

^1^Adscription region: MAG = Maghreb; EAF = East-Africa; COS = Cosmopolitan; CAN = Canary Islands; MED = Mediterranean.

^2^References are in Additional file 3.

^3^ALG = Algeria; ARP = Arabian peninsula; AZO) = Azores Archipelago; BRA = Brazil; CAN = Canary Islands; CAF = Central Africa; CAV = Cape Verde; EAF = East Africa; GYP = Gypsies; HIS = Iberoamerica; JEW = Jews; MAD = Madeira Islands; MdC = Central Mediterranean; MdE = Eastern Mediterranean; MOR = Morocco; NEA = Northeast Africa; NWE = Northwest Europe; POR = Mainland Portugal; SAM = Sahara and Mauritania; SPA = Spain; TUN = Tunisia; AUS = US Afro-Americans; CUS = Caucasian US Americans; HUS = Hispanic US Americans; WAF = West Africa.

^4^U6b = U6b'd, no U6b1, without 16174 and/or 16261; U6d = U6b'd with 16174 or 16261.

^5^Complete sequences of this study, presented in the tree (see Additional file 2) where included in subhaplogroup frequencies estimations.

### Phylogeography of U6

The large number of complete sequences analyzed allows the identification of several clusters with geographic and/or ethnic identity (Tables 2 and 3). Within U6a, sub-group U6a1 clusters together Mediterranean sequences of European or Maghreb origin. U6a2 comprises mainly of Ethiopian sequences with some outsiders. Cluster U6a8, of Maghreb expansion, shares with U6a2 and U6a3 the 16189 transition. Sub-groups of U6a3 trace multiple expansions across Europe (U6a3a), Maghreb (U6a3b and U6a3e) and West Africa (U6a3c, U6a3f). U6a5 points again to a West African spread, while U6a6 signals a radiation into the Maghreb. U6a7 is a predominantly European clade. It shows historical diffusions to the American Continent and a detectable Sephardic radiation.

**Table 2:** Geography and ages of the African and Canarian U6 sub-clades

**Haplogroup**	**Geography**	**Age**
U6		35300
U6a		26200
U6a7	Maghreb	29000
U6a7b	Maghreb	24000
U6a6	Maghreb	21900
U6a2		19700
U6a3		18800
U6a1	Maghreb	18600
U6a1b	Maghreb	17100
U6a6b	Maghreb	14500
U6d		12900
U6a2a1	Ethiopia	12700
U6b		12500
U6d3	Maghreb	10600
U6a7c	Maghreb	10600
U6c		10400
U6a8	Maghreb	8800
U6a2b	Ethiopia	8600
U6a2a1a	Ethiopia	8600
U6a5a	West Africa	8600
U6a5b	Sub-Saharan Africa	7200
U6b2	Maghreb	7200
U6a3f	Sub-Saharan Africa	6500
U6b1b	Maghreb	6500
U6c2	Maghreb	6300
U6a2a1b	Ethiopia	5900
U6a2b1	Ethiopia	5200
U6a3e	Maghreb	5200
U6a3c	Sub-Saharan Africa	3900
U6b3	West Africa	3900
U6b1a	Canary Islands	3600
U6a3b	Maghreb	2900
U6b1a2	Canary Islands	2600
U6b1a1	Canary Islands	1500
U6b1a3	Canary Islands	1300
U6c1b	Canary Islands	1300

**Table 3:** Geography and ages of the European U6 sub-clades

**Haplogroup**	**Geography**	**Age**
U6a1		18600
U6a1a		13100
U6a1a2		16200
U6a4		10600
U6a3a		9600
U6a7a		7600
U6d1		5700
U6a3a1		5600
U6a7a1		4700
U6a7a2		4200
U6a7a1c		3500
U6a7a2a1		2600
U6a1b1b	Iberia	2600
U6d1a		1700
U6a7a1b	Sephardic	1400
U6c1a	Italy	1300
U6a1a1a2		600
U6a7a1a	Acady	500

U6b is a haplogroup with low overall frequency and of uncertain origin but a wide distribution. To the East, following the Sahel corridor, it reaches Sudan and the Arabian Peninsula beyond. To the west it colonized the Canary Islands where an autochthonous lineage, U6b1a [6,7,9], appears to be a sister branch of a Maghreb expansion [8]. Northwards, U6b diffused as far as the Iberian Peninsula. Its sister clade U6d has one Ethiopian sequence as the only east African representative. The rest of U6d lineages seem to point to diffusion towards Mediterranean Europe from the Maghreb. Finally, haplogroup U6c presents two sister clades: the first, U6c1, centered in Mediterranean Europe, shows interesting contacts with the Canaries, the second, U6c2, represents another expansion in the Maghreb.

**Figure 1:**
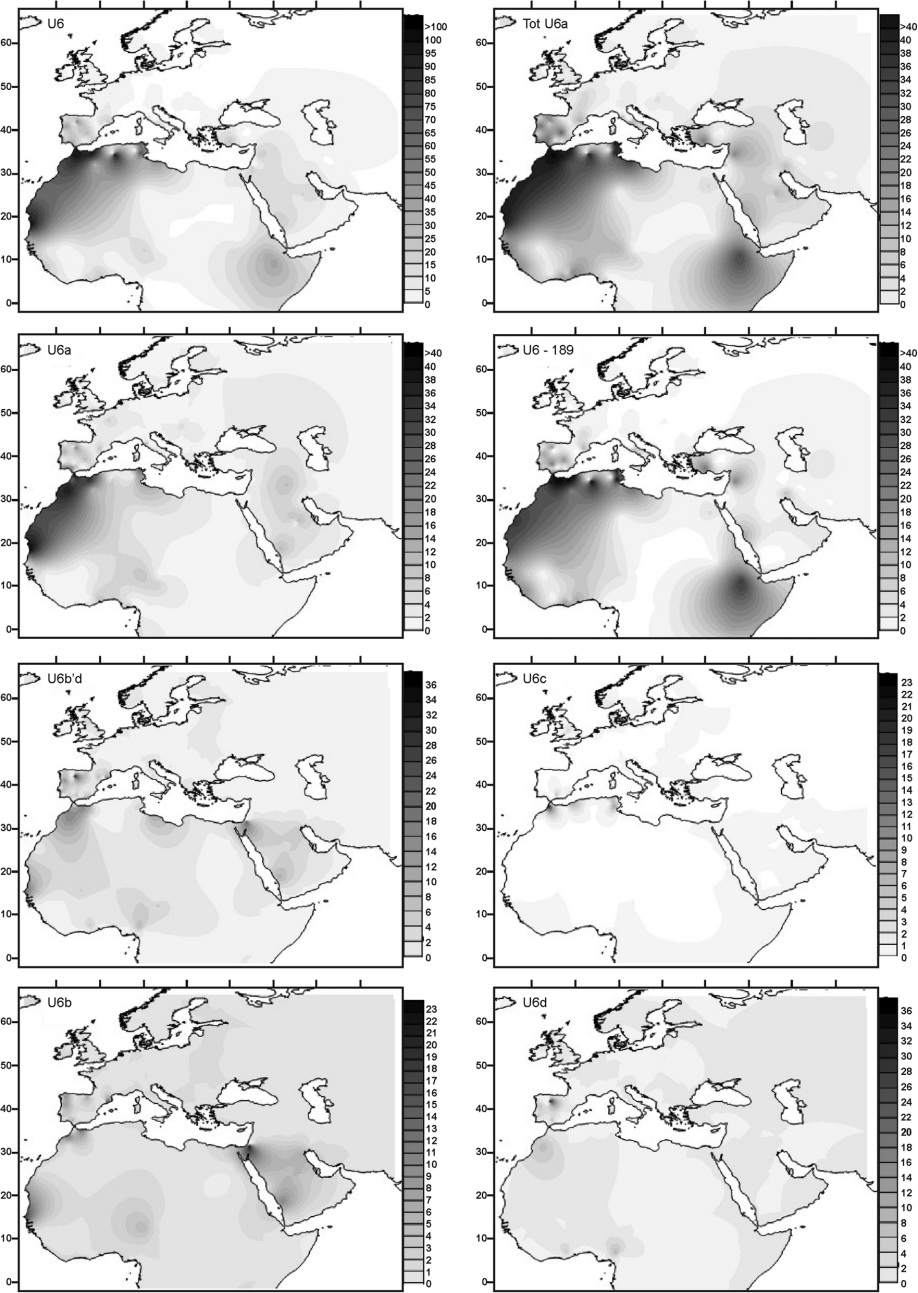
Surface maps, based on HVI frequencies (in o/oo), for total U6 (U6), total U6a (Tot U6a), U6a without 16189 (U6a), U6a with 16189 (U6a-189), U6b'd, U6c, U6b and U6d.

Although limited in its phylogenetic accuracy, the HVRI-based sequence data-set (see Additional file 4), permits a less biased analysis of the geographic diffusion of U6 lineages. Using a total of 237 sample locations, across the African Continent, Europe and the Middle East, we generated frequency maps for U6 and several sub-groups (Figure 1). The whole U6a haplogroup shows two remarkable areas of diffusion within Africa; first, the Maghreb, extending southwards through the Sahel to the Gulf of Guinea and, second, an Eastern African radiation centered on Ethiopia. The Iberian Peninsula in the West and the Levant in the East preserve signals of secondary spreads. U6a with the 16189 transition faithfully repeats the total U6a topology. However, within diffusion map of U6a, without 16189, the Ethiopian focus disappears, leaving only the West African center of dispersion.

As commented above, haplogroup U6b is widely spread at low frequency, reaching the Levant eastwards and the Sahel and Sudan belts southwards; whilst its sister clade U6d is centered in the Maghreb with punctuated spreads to Iberia and West Africa. Finally, haplogroup U6c has the most limited geographic range, extending only over the Mediterranean Maghreb with minor distributions in the Iberian Peninsula and Italy.

In order to evaluate their most probable origins, haplogroup frequency distribution patterns should be contrasted with the distribution of their respective variances. However, the number of samples with sound variances precludes their presentation as diffusion maps. For the whole haplogroup U6 and large geographic areas it is possible to estimate the respective diversities using the pi statistic. Nearly identical diversities are found for Europe (4.625 ± 0.737) and the Middle East (4.653 ± 1.230). The Maghreb (3.203 ± 0.524) and East Africa (3.097 ± 1.869) are at a second level, whilst West Africa (2.127 ± 0.961) contains the least diversity. However, the only significant differences between areas are those found when comparing Europe to the Maghreb (p = 0.036) and West Africa (p = 0.011).

### Mutation rates and calibration points calculated from Acadian pedigree

**Figure 2:**
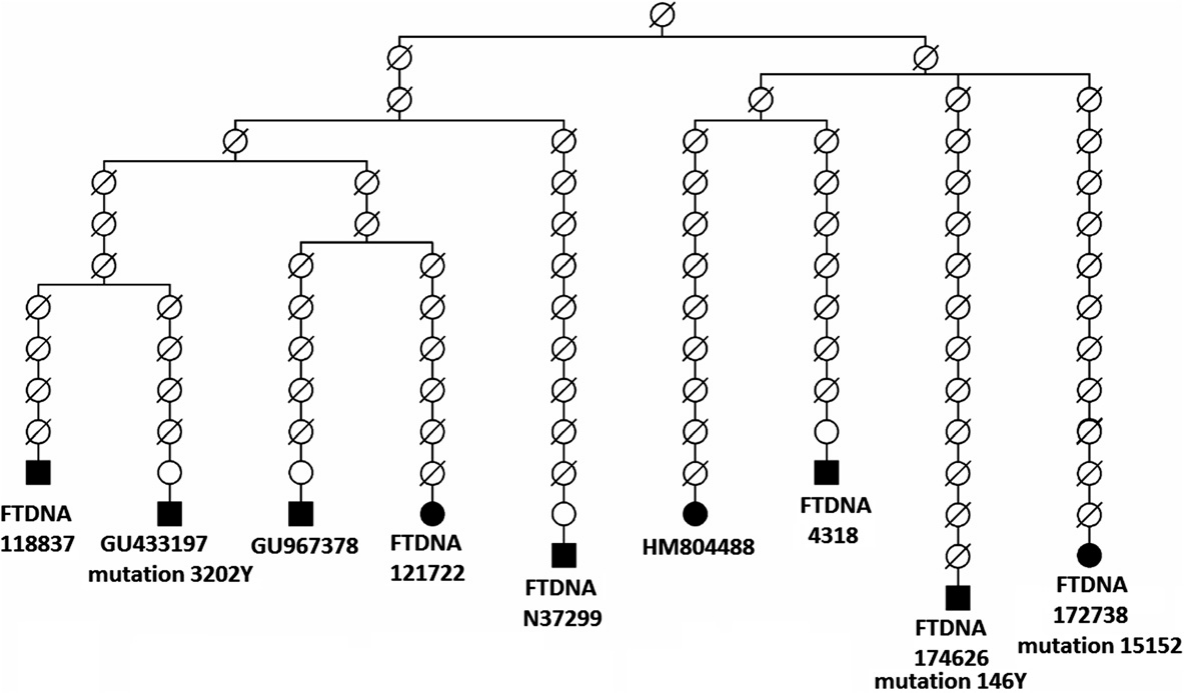
Acadian pedigree.

Nine of the eleven sequences analyzed in the Acadian cluster (U6a7a1a) come from people who are direct maternal descendants from two sisters of French origin who married in Acadia in the 17^th^ century. So we were able to build an Acadian pedigree (Figure 2), which allows us to compare phylogenetic and familial estimates of mitochondrial substitution rates. With a founding ancestor in 1625, and about 15 generations elapsed to the present, we arrive at an empirical average generation of 25 y, half-way between the 20 and 30 y generation values most commonly used [28].

We detect two heteroplasmic polymorphisms (146Y and 3202Y) and one substitution (15152) in the Acadian pedigree. Of the three polymorphisms, only 146 is a major hotspot in the mtDNA genome [23]. Site 15152 is also found in a heteroplasmic state in one sequence belonging to the Acadian cluster, which could not be included in the pedigree (see Additional file 2). Not being a fast site, it most probably represents a still segregating site, fixed in only some family members. This leave us with one substitution in 90 transmission events, giving a mutation rate of .0111 per generation (95% CI .0020-.0616), corresponding to 0.034, 0.027 or 0.022/site/My, using a complete sequence length of 16569 bp, and respective generation times of 20, 25 or 30 years.

Our pedigree mutation rate (0.034/site/My) turns out twice as high as the phylogenetic rate (0.017/site/My) [23]. Encountered on different evolutionary time scales, this discrepancy may be resolved by taking into account the probability of intra-individual fixation of mutations present in heteroplasmy, and the sex of individuals carrying a new mutation, since males will not transmit them [29,30]. We had to consider the heteroplasmic mutations as somatic because they were not found at detectable levels in other family members. However, if instead of an empirical approach, we consider the male gender bias introduced as a rule in the pedigree mutation rate estimation, and divide it by two, the pedigree and phylogenetic mutation rates will be the same, as the above authors pointed out.

Accurate sequence divergence time estimations are necessary to correlate genetic coalescence with archaeological and anthropological chronologies. Relaxed phylogenetics, based on multiple calibration points at different depth nodes, are seen as a prerequisite for appropriate dating [31], although the strength of the method depends on the availability of precise calibration points [32]. The estimation based on the Acadian pedigree could be used as a very recent calibration point.

## Discussion

### Return to Africa traced by U6

As secondary branch of the Eurasian macro-haplogroup N, phylogenetically, U6 is a non-African lineage and represents a back-migration to Africa. According to haplogroup U geographic radiation, it was suggested that the most probable origin of the U6 ancestor was in western Asia with a subsequent movement into Africa [5]. Several age estimates for the whole U6 mtDNA clade have been calculated with different sets of complete sequences, varying mutation rates and different coalescence-based approaches; including, mean pairwise distances, maximum likelihood, and internally calibrated Bayesian relaxed clock phylogenetics. Ages ranged from 33.5 ky [9] to 45.1 ky [7], but with broad credibility boundaries that largely overlap. Our own estimate of the time to the most recent common ancestor (TMRCA) for U6, using the current enlarged set of complete sequences, is 35.3 (24.6 - 46.4) ky. This period coincides with the Early Upper Paleolithic (EUP) period, prior to the Last Glacial Maximum, but cold and dry enough to force a North African coastal route.

The upper limit for the first U6 radiation within Africa, represented by the time to the MRCA of U6a is 26.2 (20.3 - 32.2) kya, and likely occurred in the Northwest 9,000 years later than the age of the whole clade. If we assume that U6 originated outside of Africa, and taking 5,000 km as an estimation of the North African coastal contour, with an homogenous coastal environment, and a simple one-dimensional diffusion model, the constant rate of advance (r) of the population carrying the U6 lineage would be 0.56 km per year, which is a reasonable value for Paleolithic hunter-gatherers [33]. Now, assuming a Paleolithic population growth rate (g) of 0.007 per year, we can calculate the migration rate (m) as 11.2 km per year using Fishers’ equation (r = 2 √(gm)). Two transitions, 3348 and 16172, separate haplogroup U6 from the basal macro-haplogroup U. Using a mutation rate of one transition in every 3,624 years [23], we estimate that an average period of about 7,000 years separates the U and U6 nodes. Although, the credible intervals of these two dates will be large, the relative placement of the two nodes should remain constant. If we place the U6 node at the northeast border of Africa, and under the same assumptions and parameters applied above, we can transform years into km, obtaining a radius of about 4,000 km outside of Africa for the place of origin of macrohaplogroup U within Eurasia.

Phylogeographic analysis using both uniparental markers repeatedly and independently pointed to the early return to Africa of modern humans after their first exodus. Focusing on mtDNA, it has been suggested that haplogroup M1 could be the travel partner of U6 [7,10]. However, there are notable differences in their geographic distributions, mainly in North Africa where U6 is predominant in the Maghreb and scarce in Egypt, while M1 shows the opposite trend, reaching its highest frequency in the latter country. The divorcing demographic histories of both haplogroups in Africa have been pointed out recently [8].

Several possible Y-chromosome counterparts of this backflow have been also described. Thus, in a phylogeographic analysis of Y-chromosome binary haplotypes [34], it was proposed that the Eurasian haplogroup R characterized by M173/M207 SNPs expanded from its origin, reaching Europe, the Middle East and India. Later it was found that a branch of this haplogroup also penetrated into Africa [11], strongly resembling the mtDNA U2, U5 and U6 trifurcation. Haplogroup T-M70, which emerged around 40 kya in Asia after the K-M9 polymorphism and has widespread but low frequency distributions in Europe and North and East Africa, has also been proposed as a signal of an ancient backflow to Africa [12,35]. Another possible signature of this Back to Africa movement could be the IJ haplogroup defined by marker M429 [36], which bifurcated early, spreading haplogroup I throughout Europe and haplogroup J through the Middle East, Ethiopia and North Africa. The ancient coalescence calculated for J1-M267 [37] further reinforces this hypothesis.

There are important differences in dating this back-migration, with mtDNA situating it in the Pleistocene [5-10] and Y-chromosome mainly in the Holocene [11-13]. This difference was previously attributed to the deeper coalescence for mtDNA compared to that for Y-chromosome lineages [38], however recent findings [39] indicates that these differences should be attributed to the fact that each uniparental markers may be detecting different gender-specific movements. On mtDNA grounds, it is known that after the Out of Africa migration around 59–69 kya, the U branch of macro-haplogroup N spread radially from somewhere in western Asia around 39–52 kya. This reached Europe, signaled by haplogroup U5, North Africa by haplogroup U6, and India by haplogroup U2 [5]. Coalescence age for U5 correlates closely with the spread of Aurignac culture in Europe and, from an archaeological perspective, it has been argued that Central Asia, not the Levant, was the most probable origin of this migration [40,41]. In absolute agreement with this vision, we propose that, in parallel, U6 reached the Levant with the intrusive Levantine Aurignacian around 35 kya, coinciding with the coalescence age for this haplogroup.

### U6 spreads into Africa

This first African expansion of U6a in the Maghreb was suggested in a previous analysis [6]. This radiation inside Africa occurred in Morocco around 26 kya (Table 2) and, ruling out the earlier Aterian, we suggested the Iberomaurusian as the most probable archaeological and anthropological correlate of this spread in the Maghreb [6]. Others have pointed to the Dabban industry in North Africa and its supposed source in the Levant, the Ahmarian, as the archaeological footprints of U6 coming back to Africa [7,9]. However, we disagree for several reasons: firstly, they most probably evolved in situ from previous cultures, not being intrusive in their respective areas [42-44]; second, their chronologies are out of phase with U6 and third, Dabban is a local industry in Cyrenaica not showing the whole coastal expansion of U6. In addition, recent archaeological evidence, based on securely dated layers, also points to the Maghreb as the place with the oldest implantation of the Iberomaurusian culture [45], which is coincidental with the U6 radiation from this region proposed in this and previous studies [6]. In the same publication, based on partial sequences [6], we also suggested a migration from the Maghreb eastwards to explain the Ethiopian radiation but, in the light of complete sequence information, it seems that it was an independent spread [9]. In the present study, the U6a2 branch shows an important radiation centered in Ethiopia (Table 2) at around 20 kya (see Additional file 2). However, this period corresponds with a maximal period of aridity in North Africa and a return to East Africa across the Sahara seems unlikely. The most probable scenario is that small human groups scattered at a low density throughout the territory, retreated in bad times to more hospitable areas such as the Moroccan Atlas Mountains and the Ethiopian Highlands. Given the still limited U6 information from Northeast African and Levant populations, we are unable to hypothesize the route followed by the U6 settlers of Ethiopia and to correlate them to an appropriate archaeological layer. In this respect, the absence of U6 representatives in autochthonous populations from Egypt [46-48] and its scarcity in cosmopolitan samples [49,50] is puzzling. However, our model has an important outcome. It is that the proposed movement out of Africa through the Levantine corridor around 40 kya did not occur or has no maternal continuity to the present day. This is because: first, in that period the Eurasian haplogroups M and N had already evolved and spread at continental level in Eurasia, and, second, there is no evidence of any L-derived clade outside Africa with a similar coalescence age to that proposed movement. Under this perspective, the late Pleistocene human skull from Hofmeyr, South Africa, considered as a sub-Saharan African predecessor of the Upper Paleolithic Eurasians [51], should be better considered as the southernmost vestige of the *Homo sapiens* return to Africa. The knowledge of its mtDNA and Y-chromosome affiliations would be an invaluable test for our hypothesis. The rest of the human movements inside Africa, such as the Saharan occupation in the humid period by Eastern and Northern immigrations, or the retreat to sub-Saharan African southwards and to the Maghreb northwards in the desiccation period [52], or even the colonization of the Canary Islands, all faithfully reflect the scenarios deduced from the archaeological and anthropological information.

Around the same period of 20 kya, other U6a branches radiated within the Maghreb (U6a3, U6a6, U6a6b, U6a7, and U6a7b), with possible spreads to the Iberian Peninsula (U6a1, U6a1b). However, from 17 kya to 13 kya there was a notable population stasis, as lineage expansions are not detected (see Additional file 2). After that, the climate shifted to a humid period in Africa and population growth was reinitiated. In Ethiopia, periodical bursts at around 13 kya (U6a2a1), 9 kya (U6a2b, U6a2a1a) and 6 kya (U6a2a1b) are detectable (Table 2).

Basic clusters like U6b, U6c and U6d also emerged within a window between 13 to 10 kya (Table 2). U6b lineages spread from the Maghreb, through the Sahel, to West Africa and the Canary Islands (U6b1a), and are also present from the Sudan to Arabia, but not detected in Ethiopia. In contrast, U6c and U6d are more localized in the Maghreb. Further spreads of secondary U6a branches are also apparent, going southwards to Sahel countries and reaching West Africa (U6a5a). Autochthonous clusters in sub-Saharan Africa first appeared at around 7 kya (U6a5b), coinciding with a period of gradual desiccation that would have obliged pastoralists to abandon many desert areas [52]. Consequently, no more U6 lineages in the Sahel are detected, while later expansions continued in West Africa (U6a3f, U6a3c, and U6b3) and the Maghreb with an additional spread to the Mediterranean shores of Europe involving U6b2, U6a3e, U6a1b and U6a3b1.

**Figure 3:**
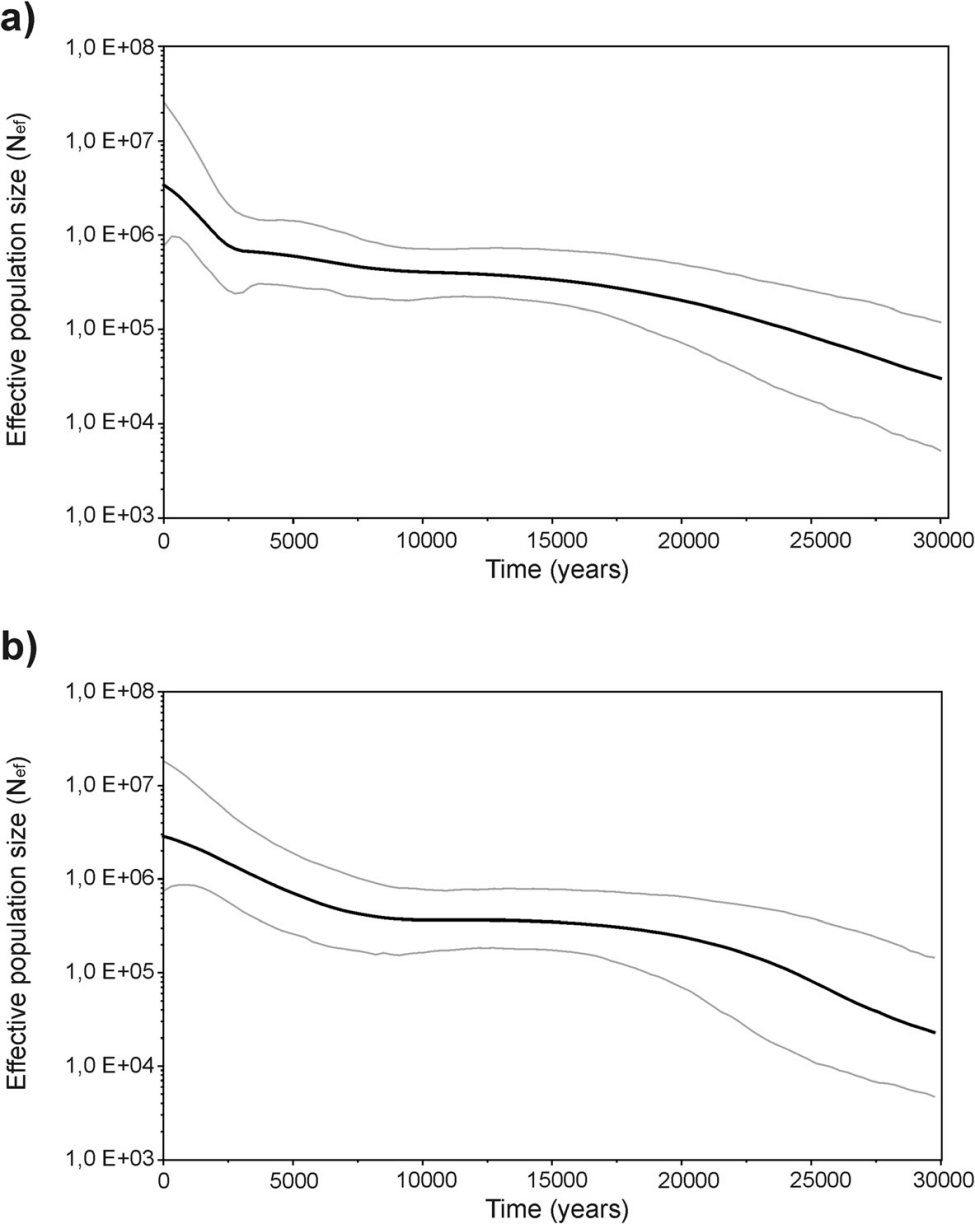
Bayesian Skyline Plots (BSPs) of effective population size (thousands) through time (ky BP) inferred from U6 total (a) and African (b) complete sequences.

In principle, these demographic events deduced by direct lineage inspection are better modeled using coalescence theory to estimate past population size [53]. A plot of population size through time using the complete set of U6 sequences (Figure 3a) shows a gradual expansion to around 15 kya, followed by population stasis until 3 kya when a second expansion began and extended to the present. However, this pattern seems in contradiction with the expansions and stasis observed for Africa in the U6 tree as commented above. As the total set of sequences include European sequences, sometimes grouped in European clusters, we wonder whether the population dynamics could be different in the two continents. Consequently, we repeated the analysis using only African sequences (Figure 3b). The inferred demographic pattern then fits better with the paleo-climatic fluctuations proposed for North Africa: population grew moderately until the Last Glacial Maximum around 20 kya and showed a 10 ky stasis until the African wet period starts, coinciding with early Neolithic. Then a second growth is observed until nowadays. The dry period that desiccated the Sahara and Sahel around 5 kya is not detectable in the plot. However, this apparent anomaly could be justified for at least two reasons: first, populations continued expanding to Mediterranean and sub-Saharan borders; second, cultural improvements made human populations less susceptible to climatic fluctuations.

The subdivision of HVI sequences into geographic components (Table 1) shows that the Maghreb component is dominant over all of North Africa, reaching 45.7% even in Arabia. Frequencies drop in Central and West Africa, suggesting a southward spread, and it is absent in East Africa where all haplotypes belong to the Ethiopian U6a2 cluster. This East African lineage is also the most prevalent in Central and West Africa, pointing to a westward expansion through the Sahel corridor. In North Africa it is second in frequency except in Algeria where it is dominant (55%).

As there are no obvious geographic gradients, the analysis of the geographic components indicate that U6a2 may have reached the region through the Sahara, by maritime contacts from the Levant or, most probably both. U6c is confirmed to be a Maghreb lineage restricted to the Mediterranean area. It is also confirmed that U6b has the most widespread geographic range. However, haplotypic matches occur only between geographically continuous regions, in the west linking the Maghreb up to Atlantic Europe and down to the Canaries and West Africa, and in the east the Levant with the Arabian Peninsula. Its absence in East Africa makes the search for its origin and dispersion routes difficult. In any case, its present-day western and eastern areas must have been connected sometime in the past, perhaps through the Sahara during the Holocene Humid Period.

### The colonization of the Canary Islands

This archipelago is only 100 km from the Western Sahara. When discovered by the Europeans in the 15^th^ century, it was inhabited by indigenous people, today collectively known as Guanches. On the basis of anthropological, archaeological and linguistic grounds, close affinities with the North African Berbers were soon identified [54]. Molecular analyses have confirmed these affinities. In fact, two mtDNA Canary autochthonous U6 subgroups, U6b1a (16163) and U6c1 (16129) were proposed as signals of their relatedness with North African populations [6].

Later studies of indigenous remnants confirmed that these lineages were in the Canaries before the European colonization [55,56]. Although the majority of the 14C data are under suspicion, it is broadly accepted that the most ancient human settlement on the Canaries was not earlier than 2.5 kya [54]. This contrasted with the first estimated age for U6b1a of 5.8 ± 4.5 kya using a set of 45 HVI sequences [6]. A new estimation, based on complete sequences dated the clade to about 2.9 (2.1; 3.7) kya [23]. However, when the archaeological date for the colonization of the Canary Islands was used as a calibration point in a U6 Bayesian phylogenetic analysis based also on complete sequences, the U6b1a age estimation was 4.8 (2,9-7.1) kya [9]. The age for another potential founder clade H1 (16260) was also estimated at 6.3 ± 2.9 kya, much older than the archaeological date [55]. To reconcile these discrepancies, it was suggested that more than one founder haplogroup lineage arrived on the islands. This was based on two unexpected results: first, the high diversity found among the aboriginal samples, at the same level as current populations and second, the detection of basic and derived U6b1a and U6c1 haplotypes in the aborigine remnants ([55,56] and unpublished results). So, at least the basic U6b1a haplotype (16163, 16172, 16219, 16311) and three derived ones with respectively 16048, 16067 and 16092 additional transitions, the basic U6c1 haplotype (16129, 16169, 16172, 16189) and a derived one with the additional 16213 transition were on the islands before the European colonization. Focusing on complete sequences (see Additional file 2), three putative Canary Islands U6b1a subgroups are distinguishable: U6b1a1 (7700), U6b1a2 (6734) and U6b1a3 (15697, 16092) with ages of 1,546 (0–3.3), 2,585 and 1,287 ya respectively, and a putative Canary U6c1b (16086) subgroup with 1,287 (Table 2), the same age as U6c1a, a putative southern Italian clade (Table 3). It has also been possible to calculate coalescences of U6b1c and U6c1b based on HV1 sequences, giving ages of 1,906 (38–3774) and 2,085 (2,001-6,170) years respectively. All these subgroup dates are better conciliated with the archaeological estimations.

Another unsettled question about the aboriginal colonization of the Canary Islands is whether they arrived in one or several waves. It is now known that U6c1 (16129) cannot be considered a Canary autochthonous lineage. In addition to the Canaries, two southern Italians, one Andalusian from Cordoba (see Additional file 2), and one Sened Berber from Tunisia were also detected [57]. All these focus its origin in the Mediterranean area in Roman or Arab times. The presence of U6c1 female lineages in the Canaries suggests a premeditated maritime colonization of the islands, not only a sporadic male contact. Surprisingly, no U6b1a counterpart had been found on the African continent. In principle, this should not be a surprise as U6b seems to be a residual haplogroup that had a wide expansion in the past but very low frequencies at present. However, in a recent article [8], a Canary specific U6b1a branch was further refined because two (9738 and 15431) of the four mutations that defined this lineage were shared by U6b1b sequences found in the Maghreb relating the Canary lineage origins, as in the case of U6c1, to this North African area. So, we can guess that the arrival of this lineage occurred within a window from 2.6 to 1.3 kya, also in Roman or Arab times and with similar geographic origins as U6c1. By parsimony, this would favor a sole colonization wave for the Canaries, although several waves from the same area are also possible. The fact that, even in the present day population of the Canaries, U6c1 is significantly more frequent in the eastern islands of Gran Canaria, Fuerteventura and Lanzarote [58] and the high genetic diversity found in the aboriginal colonizers of Tenerife and La Palma [6,55] seem to favor the several waves alternative. Curiously, one U6b1 lineage has been sporadically detected in a Lebanese mtDNA survey that might bring speculation about a Levantine origin for the U6b1 cluster [59]. However, a more or less recent immigration of this lineage from the Canary Islands seems more convincing explanation.

### Reaching Europe

In general, haplogroup U6 has very low frequencies in Europe. It is more frequent in the Mediterranean countries, mainly in those with longer histories of Moorish influence since medieval times, such as Portugal (2.5%), Spain (1.1%) or Sicily (0.4%). In fact, there is a significant longitudinal gradient in Mediterranean Europe, with frequencies decreasing eastwards (r = −0.87; p = 0.008) that run parallel to that found in North Africa (r = −0.97; p < 0.001). Congruently, the presence of U6 in the Iberian Peninsula has been attributed to the historic Moorish expansion [60]. However, without denying this historic gene flow, others have also suggested prehistoric inputs from North Africa [61].

Actually, the U6 phylogeny and the phylogeography of its lineages are better explained admitting both prehistoric and historic influences in Europe. Traces of Paleolithic and early Neolithic presence of U6 in Mediterranean Europe are the two Iberian lineages at the root of the U6a1 expansion of 18.6 kya, without involving any North African counterpart (Table 3). Again, when the next U6a1a radiation occurred at 13.1 kya, a lineage later expanded at its node as the U6a1a2 clade and only led to European sequences. There are also two sequences of Mediterranean European origin that directly emerged from the ancestral node of the East African cluster U6a2a (19.8 kya). The presence of a third Mediterranean European sequence identical to a Tunisian one that coalesces with a Palestinian sequence about 5.9 kya suggests that these eastern lineages most probably reached Italy, Iberia and the Maghreb from the Levant through maritime contacts since the Neolithic. Another Italian sequence that coalesces at 10.6 kya with a Levantine sequence forming the U6a4 clade reinforces such a conclusion. More difficult to ascertain is the presence of 3 additional Italian sequences that directly sprout from the basal node of the west sub-Saharan African clade U6a5 (12.7 kya). There are two clusters, U6a3a (9.6 kya) and U6a7a (7.6 kya), with mostly European sequences, that expanded in Neolithic times. Other European groups: U6a3a1, U6a7a1, U6a7a2, and U6c1 spread within the Chalcolithic period. Finally, at least 14 European lineages have coalescence ages in historic times. Some may be associated with the Roman conquest of Britain (U6d1a), the diaspora of Sephardic Jews (U6a7a1b), or the European colonization of the Americas (U6a1a1a2, U6a7a1a, U6a7a2a1, U6b1a). Roughly, 35 European lineages have prehistoric spreads and 50 sequences historic spreads. In all cases they are involved with clear North African counterparts.

With less accuracy, information from HVI sequences also provides a phylogeographic perspective of U6 in Europe (Table 1). The largest U6 Maghreb component in Europe is found in Portugal (69.9%), then in Spain (50.0%) and Italy (53.0%), and decreases sharply in the Eastern Mediterranean (25.0%). No U6b representatives have been detected in Italy, although it is present in Iberia to the west and in the Near East to the east. Regarding the Canarian motif, 33% and 50% of the U6b haplotypes found respectively in mainland Portugal and Spain belong to the Canary Islands autochthonous U6b1a subgroup. Curiously, it has not been detected in the Portuguese island of Azores and Madeira or in Cape Verde either [58]. U6c is confirmed as a low-frequency Mediterranean haplogroup. All four identified U6 HVI components have representatives in Atlantic Europe. This Maghreb component could have arrived through Atlantic Copper or Bronze age networks, leaving the presence of U6c to Punic or more probably, Roman colonization.

On the other hand, the East African component in Europe has its peak in eastern Mediterranean area (62.5%) and gradually diminishes westward toward Italy (46.0%), Spain (28.3%) and mainland Portugal (20.0%). Complemented with the previous phylogeographic information obtained from complete sequences, it seems that the Levant component points to maritime contacts from the Neolithic onwards. Congruently, archaeological comparisons of the different prehistoric cultures that evolved on both shores of the Mediterranean Sea point to the conclusion that each region had its own technological traditions, despite some parallel developments. This finding weakens the hypothesis of important demic or cultural interchanges, at least until the beginning of the Neolithic when prehistoric seafaring started in the Mediterranean Sea [62]. Indeed, the rapid spread of the Neolithic Cardial Culture, or the presence of the Megalithic culture on both sides of the Mediterranean during the Chalcolithic period, would suffice to explain the presence in Europe of U6 lineages with coalescence ages since Neolithic times onwards. However, at least two U6 lineages, U6a1a and U6a5, both with European coalescences around 13 kya, are left devoid of archaeological support. These would coincide with climatic improvement during the Late Glacial period. Curiously, several European mtDNA lineages, with similar coalescence ages, such as V [63], U5b1 [64], H1 and H3 [65-67], have been proposed as maternal footprints in North Africa of a hypothetical southward human spread after the Last Glacial period, from the Franco-Cantabrian refuge. This also lacks archaeological evidence. Accurate phylogeographic analysis of these and other mtDNA and Y-chromosome haplogroups are needed to disentangle these puzzling patterns.

### U6 in the Jews

There are 15 complete U6 sequences in our tree that are recognized to belong to the Jewish community. Six of them are grouped into a Sephardic cluster U6a7a1b of diverse geographic sources with another five sequences of possible Jewish maternal descent. This wide spread testifies to the extent of the forced exile of this community of Hispanic origin. As a rule, the rest of the sequences are included in haplogroups that match their geographic origins. Thus, 2 Moroccans and 1 Tunisian respectively belong to Maghreb haplogroups U6a1b and U6a7a1, 2 Bulgarians and 1 Turk are included in different branches of the mainly Mediterranean haplogroup U6a3 and 1 Ethiopian merges into the East African U6a2a1b clade. However there are two exceptions, 1 Russian has a sequence at the same level as the East African cluster U6a2, and 1 Ethiopian belongs to the Mediterranean clade U6d2. Except for the Sephardic subgroup, all these Jewish sequences are isolated branches in their respective haplogroups with no close relatives.

From a sample of 2,860 HVI Jewish sequences, only 15 (0.5%) were classified as U6 (Table 1). The Maghreb component captures 26.7% of them and the East African component, the remaining 73.3%. The bulk of the sequences therefore seem to have their origin in the Near East.

### U6 in the Gypsies

None of the complete sequences has been attributed to Gypsy origin, and only 7 HVI sequences from a sample of 944 Gypsies (0.7%) turned out to be U6. Three of them (43%) are of Maghreb origin and the other four (57%) belong to haplogroup U6b. As the Gypsies originate in India, where U6 is practically absent, they must have acquired these maternal lineages by admixture with Mediterranean populations during their long migratory history.

### U6 participation in the New World colonization

Pair-wise genetic distances based on only one genetic marker may not show the true relationships between populations, due to confounding drift or selective effects. However, looking at the geographic partition of the U6 lineages that reached the New World with the European colonists, the origin of this maternal gene flow can be ascertained in most of the American samples studied.

**Figure 4:**
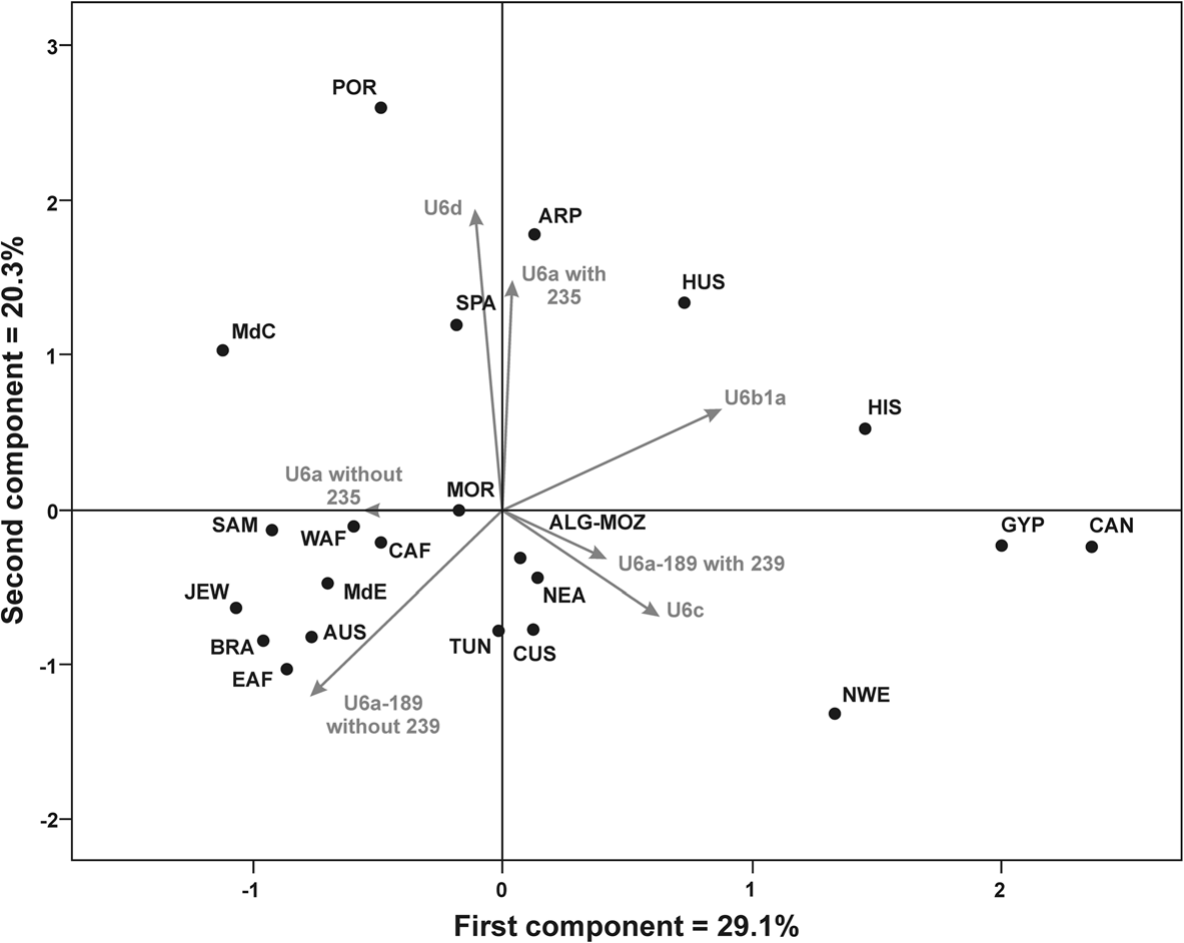
**Principal Component Analysis (PC) based on U6 subhaplogroup frequencies in the different populations studied.** The position occupied by each sub-haplogroup is indicated in gray.

a) *The U6a7a1a Acadian cluster from Canada:* Male French colonists arrived in the Canadian region of Acadia at the beginning of the 17th Century. However, the core group of maternal lineages that gave rise to the French Acadian population did not settle in the area until the middle of that century (http://www.acadian-home.org/). At least one of those maternal lineages belongs to the sub-haplogroup U6a7a1a, defined by mutations 2672 and 11929. Putative descendants of that lineage are represented by 11 complete extant French-Canadian sequences in our U6 tree (see Additional file 2). Applying the recently proposed overall mtDNA mutation rate [23], we obtain a mean phylogenetic age of 467 years for this cluster, in close agreement with its history. Another closely related sequence, which lacks the Acadian basal substitution 2672 (see Additional file 2), roots the cluster’s ancestor in France around 3,000 ya in the late European Bronze age.

b) *Diverse geographic origins for the United States U6 sequences:* As a result of geographically different gene flows, the US population is ethnically diverse and so its U6 lineages would be expected to have different origins. Indeed, focusing on complete sequences (see Additional file 1), there are 19 of US origin or most probably so (Sequences EF 657375 and EF 657774). Three of them are grouped together, conforming a US cluster (U6a1a1a2) with a coalescence age around 600 ya, having another USA lineage and North African and European Mediterranean sequences as sister clades. Five are found within a mainly sub-Saharan Africa background (U6a3c, U6a5). Six have European sequences as their closest relatives but with Maghreb ancestors, of them four have UK (U6a7a1, U6a7a2a), one has French (U6a1b1a) specific provenance, and the other one directly groups with an Iberian lineage (U6a3a2). For the remaining four, two are found within a Maghreb cluster (U6a7c), and two root with a Maghreb sequence within an European cluster (U6a3a1). Information gathered from HVI sequences (Table 1) allows a more precise quantification of the origin and distribution of U6 in the US. Although this haplogroup has frequencies less than 1% in the three main ethnic communities: US Afro-Americans (AUS) (0.62%), Caucasian US Americans (CUS) (0.31%), and Hispanic US Americans (HUS) (0.75%), their U6 geographic components are different. AUS shows the highest East African component (78.6%), a moderate contribution from the Maghreb (21.4%) and lacks U6b and U6c lineages. This distribution suggests that the bulk of U6 in AUS was not brought by the transatlantic slave trade in sub-Saharan West Africans but by significant later voluntary migration from East Africa. CUS has more evenly balanced frequencies of the Maghreb (44%) and East African (50%) components that mimic those in Italy and Atlantic Europe, their most probable contributors. In addition, its U6b (6%) component is not of Canarian origin. On the contrary, for HUS, U6b (62.5%) lineages are the most frequent and 60% of them belong to the native Canary Island U6b1a subgroup. This strongly supports their Spanish American origin and the relatively important role that the Canary Islanders played in the colonization of the Americas.

c) *U6 in the Iberian colonization of America:* There are only four complete sequences with Spanish American origin in our tree (see Additional file 1). Two of them are included in U6a7a1b, a Sephardic Jewish cluster. The other two are from Cuba but with maternal Canary Islands ancestors, as both belong to the autochthonous U6b1a subgroup. There are 8 Brazilian and 29 Spanish American U6 sequences in our HVI data-set, representing a frequency around 0.6% in both cases (Table 1). Brazilians lack U6b and U6c representatives and show a prominent East African component (87.5%). This contrasts with the Portuguese, the main European colonizers of Brazil (Table 1), who present high frequencies for Maghreb (69.9%) and moderate (20.0%) for East African components. However, the U6 profile of Brazilians closely corresponds to that of the Jews (Table 1). It is well known that Sephardic Jews settled in Brazil since the beginning of its colonization, mainly due to persecution by the Inquisition [68]. Congruently, Cape Verde, also colonized by Portuguese, has an important Y-chromosome Sephardim influence [69,70] and also the most prevalent U6 Eastern African component (70.0%) in Macaronesia Islands. In turn, Spanish Americans have a U6 partition more similar to the Canary Islands than to Spain, mainly due to their high frequencies for haplogroups U6b (65.7%) and U6c (5.7%). In fact, 96% of these lineages are autochthonous to the Canaries. Taken the frequency of U6 there (16.2%) we can tentatively infer that the maternal contribution of the Canary Islanders to the American colonization was around 4%.The origin of the American U6 lineages is graphically reflected by their relative positions with respect to its most probable Old World source in the PCA plot shown in Figure 4. Paying attention to the first component, the Canary autochthonous U6b1a subgroup pulls these islands and samples possessing this subclade [such as HIS (Iberoamerica), HUS (Hispanic US Americans), SPA (Spain) and POR (Portugal)] to the right. Other samples harboring other U6b related subgroups also approach this conglomerate [ARP (Arabian Peninsula), NWE (Northwest Europe), ALG (Algeria), and GYP (Gypsies)]. Those samples with an important East African component (U6a with 16189 and without 16239) are clustered on the left, as are the parental EAF (East Africa) and the JEW (Jews), AUS (US Afro-Americans), and BRA (Brazil) deriving from it. The second component further separates those samples with an important Maghreb component in Africa, like TUN (Tunisia), MOR (Morocco), WAF (West Africa), NEA (Northeast Africa), SAM (Sahara and Mauritania) and CAF (Central Africa), pulling with them those Mediterranean areas under its influence: MdC (Central Mediterranean), MdE (Eastern Mediterranean) and secondary migrants in North America like CUS (Caucasian US Americans).

## Conclusions

Complete genome sequencing, accompanied by complex statistical analysis will model the future of population genetics. However, the coalescent and phylogeographic power of uniparental markers will continue to offer a fine temporal and spatial dissection of past human movements, susceptible to be contrasted with archaeological and anthropological records. This has been the ultimate goal of this U6 study and those preceding it [6-9]. Thus, fluctuating population size inside Africa inferred from the U6 phylogeny faithfully reflect the climatic changes that occurred in this Continent affecting also the Canary Islands. Mediterranean maritime contacts drove these lineages to Europe, at least since Neolithic times. In turn, the historical European world-wide colonization brought different U6 lineages throughout the American Continent leaving there the specific sign of the colonizers origin.

## Availability of supporting data

The new complete mitochondrial DNA sequences are registered under GenBank accession numbers: JX120708-JX120776. All data from this publication are available from the Dryad Digital Repository: http://dx.doi.org/10.5061/dryad.q2h0c Data files: Secher et al.

## Competing interest

The authors declare no conflict of interest.

## Authors’ contributions

BS, JML, VMC and AMG conceived and designed the experiments. RF, JML, VMC and JJP performed the experiments. BS, RF, JML, VMC, and AMG analyzed the data. BS, RF, JML, VMC, PE, JJP, AMG contributed reagents/materials/analysis tools. BS, RF, JML, VMC and AMG wrote the draft manuscript. BS, RF, JML, VMC, PE, JJP, AMG participated in the discussion of the data and wrote the paper. All the authors read and approved the final manuscript.

## Supplementary Material

Additional file 1 **Accession numbers, subhaplogroup assignation and maternal origin for the 40 U6 lineages sequenced in the present work, the 29 lineages obtained from U6 FTDNA members contacted trough FTDNA, for 160 U6 sequences available from GenBank, and for 1 U6 sequence from literature **[71]**.** Click here for file

Additional file 2 U6 phylogenetic tree based on 230 complete sequences. Click here for file

Additional file 3 **References cited in Table **1**.** Click here for file

Additional file 4 **Geographic frequency of U6 (‰), and subgroup lineages (%).** Hyphens indicate that information about U6 sublineage classification is not available. Click here for file
